# Spatial Distribution of HIV Prevalence among Young People in Mozambique

**DOI:** 10.3390/ijerph17030885

**Published:** 2020-01-31

**Authors:** Rachid Muleia, Makini Boothe, Osvaldo Loquiha, Marc Aerts, Christel Faes

**Affiliations:** 1 Interuniversity Institute for Statistics and Statistical Bioinformatics, Hasselt University, 3590 Diepenbeek, Belgium; marc.aerts@uhasselt.be (M.A.); christel.faes@uhasselt.be (C.F.); 2 Department of Mathematics and Informatics, Faculty of Sciences, Universidade Eduardo Mondlane, 254 Maputo, Mozambique; osvaldo.loquiha@gmail.com; 3 Department of Public Health and Primary Care, Faculty of Medicine and Health Sciences, Ghent University, 9000 Ghent, Belgium; makini.boothe@gmail.com

**Keywords:** HIV/AIDS, generalized geoadditive model, kriging mixed model, Mozambique

## Abstract

Mozambique has a high burden of HIV and is currently ranked sixth worldwide for adult prevalence. In Mozambique, HIV prevalence is not uniformly distributed geographically and throughout the population. We investigated the spatial distribution of HIV infection among adolescents and young people in Mozambique using the 2009 AIDS Indicator Survey (AIS). Generalized geoadditive modeling, combining kriging and additive modeling, was used to study the geographical variability of HIV risk among young people. The nonlinear spatial effect was assessed through radial basis splines. The estimation process was done using two-stage iterative penalized quasi-likelihood within the framework of a mixed-effects model. Our estimation procedure is an extension of the approach by Vandendijck et al., estimating the range (spatial decay) parameter in a binary context. The results revealed the presence of spatial patterns of HIV infection. After controlling for important covariates, the results showed a greater burden of HIV/AIDS in the central and northern regions of the country. Several socio-demographic, biological, and behavioral factors were found to be significantly associated with HIV infection among young people. The findings are important, as they can help health officials and policy makers to design targeted interventions for responding to the HIV epidemic.

## 1. Introduction

Mozambique is among the 10 countries most affected by HIV in the world, with the world’s sixth highest prevalence among adults aged 15–49 [1]. In 2009 the HIV prevalence in adults was estimated as 11.5%, with a prevalence of 13.1% for women as compared to a prevalence of 9.2% for men [2]. A more recent AIS reported the HIV prevalence in the adult population as 13.2%, where the HIV prevalence among women was estimated at 15.4% and 10.1% among men within the same age group [3]. The HIV epidemic in Mozambique is not uniformly distributed throughout the population. Adolescents and youth aged 15–24 are one of the most vulnerable populations, with an estimated prevalence of 7.9% in 2009 and 6.9% in 2015 [2,3]. Moreover, in this age group, women account for a disproportionate number of infections, with a prevalence (11.1%) three times higher than that of their male peers (3.7%). The epidemic also shows substantial variation throughout the country, with the prevalence among this age group ranging from 2.9% in Niassa province, in the north, to 13.1% in Sofala province, in the center of the country [2].

There are many potential factors that position adolescents and young people at high risk of HIV. A recent study conducted in Maputo City points to low educational level and early sexual debut as important factors contributing to high HIV prevalence among youth [4]. Furthermore, Dias et al. [5] mention numerous biological, socio-economic, and socio-cultural characteristics that are linked to higher HIV prevalence among women in Mozambique, including age, sex of household head, wealth status, and marital status. Although the knowledge of biological, socio-economic, and socio-cultural drivers of the HIV epidemic is quite extensive, there is still a knowledge gap concerning the geographical distribution of HIV/AIDS across the country. Simply put, HIV/AIDS prevalence is not only influenced by socio-demographic, biological, and sexual behavioral factors; it can also differ significantly across districts and regions. Therefore, a thorough understanding of the geographical variability of HIV/AIDS is of paramount importance as it will help health officials formulate targeted interventions and identify where to prioritize the allocation of limited resources [6].

In this paper, we seek to understand the spatial distribution of HIV/AIDS in Mozambique among adolescents and youth by using generalized geoadditive models. The rationale for the application of this type of model is that spatial information is generally regarded as a surrogate of unobserved risk factor which may appear hard to quantify [7,8]. Our approach is an extension of the model introduced by Kammann and Wand [9]. Vandendijck et al. [10] proposed to estimate the range parameter in this model, instead of fixing it. The novelty of our approach lies in extending the estimation method of the range parameter in the context of a binary outcome. In addition, our method takes the survey design and the sampling frame into account. The structure of the paper is as follows. Section 2  gives a description of the data, a brief discussion on traditional methods to analyze geostatistical data, the statistical methods used, and details on the translation of the estimation method proposed by Vandendijck et al. [10] to the binary setting. Section 3 summarizes the results, comparing the estimates under different covariance functions and identifying the risk factors that are related to HIV prevalence in young people. We close the manuscript with a discussion including conclusions and opportunities for further research.

## 2. Methodology

### 2.1. Data Description

To study the spatial distribution of HIV prevalence and the risk factors associated to HIV infection among young people aged 15–24 years in Mozambique, we used data from the first population-based nationally representative survey on HIV sero-prevalence, AIS 2009, which is publicly available for secondary research at https://dhsprogram.com/. Ethical approval for the survey was obtained from the National Bioethics Committee for Health (CNBS) in Mozambique and the Centers for Disease Control and Prevention (CDC) and ICF Macro in the USA [2]. The Mozambique AIS 2009 is a complex survey with a stratified multistage cluster sampling design that collected a variety of information on knowledge, attitude, and risk behavior related to HIV infection; socio-demographic and cultural factors; fertility, marriage, and sexual activity; testing and counseling in health, etc. The information was collected from four questionnaires: a household questionnaire, an individual questionnaire for young people and adults aged 15–64 years, an individual questionnaire for adolescents aged 12–14 years, and an individual questionnaire for the parents or guardians of children aged 0–11 years. Moreover, the survey collected geographic coordinates of the enumeration areas where households were sampled. Further information related to the sampling process and how the survey was conducted can be found in the Mozambique AIS 2009 final report [2]. The analysis in this study was limited to a sub-sample of 2589 adolescent and young people, aged 15–24 years, who consented to being tested for HIV. For each sampled individual, a sampling weight is available, representing the complex sampling scheme.

HIV sero-status was the outcome variable used to investigate the spatial distribution of HIV infection and the factors associated to it, coded as one if the individual tested positive, and zero if the individual tested negative. To determine the HIV sero-status of the individuals, finger-prick blood spot specimens were taken from all individuals who consented to biological testing for HIV. Blood specimens were tested for HIV using two sequential enzyme-linked immunosorbent assay (ELISA) tests. Reactive blood specimens in the first test of the sequence (Vironostika) were then submitted to a second confirmatory test using Murex. If a reactive sample in Vironostika was found to be negative in Murex, a third test was used for confirmation (GeneScreen). More details on the testing algorithm are described elsewhere [2].

The explanatory variables considered in this study can be grouped into socio-demographic factors, biological factors, and HIV knowledge and behavioral factors. The socio-demographic factors were age, sex of the respondent, educational level, occupation, marital status, sex of the head of household, wealth status, mass media exposure, religion, and type of place of residence (urban, rural). Biological factors comprised variables such as STIs in the last 12 months and blood transfusion. HIV/AIDS knowledge and attitudes included awareness, stigma/prejudice, previous HIV testing, and knowing someone with AIDS. Behavioral factors included alcohol use, multiple sexual partners, sexual debut, and condom use. A detailed description of the variables used in this study is presented in Table A1. Variables such as media exposure, HIV/AIDS awareness, and HIV/AIDS stigma are composite indices derived through principal component analysis (PCA) using a set of correlated variables. The summary indices for each dimension were constructed based on the first component scores. The PCA scores were divided into tertiles following a similar approach to Magadi and Desta [11] and Dias et al. [5].

### 2.2. Generalized Geoadditive Model

Kriging is a common strategy for analyzing and understanding point-reference data, also known as geostatistical data. This technique makes predictions at one or more non-observed locations from a collection of data observed at *n* sampled locations. The most popular kriging model assumes that the underlying process is a Gaussian process. The Gaussian assumption, generally imposed in the underlying process, is often not appropriate, as the observed process might be continuous though highly skewed, or related to counts or dichotomous data. Classical geostatistical approaches such as log-normal and indicator kriging are often recommended for non-Gaussian data [12,13]. However, resulting estimates from these approaches are difficult to interpret and are also subject to bias induced by the back-transformation [14]. Additionally, kriging itself does not allow study of the effect of possible risk factors on the outcome variable, as high prevalence in a region might be linked, for instance, to lower education level and other behavioral factors. Universal kriging is another extension, allowing for the inclusion of covariates. However, it can only accommodate for linear effects. For a continuous response, Kammann and Wand [9] introduced a geoadditive model, which is a combination of an additive model that accounts for non-linear effects and a kriging model that accounts for spatial dependence.

To study the spatial distribution of HIV prevalence among young people across the country, we used a generalized geoadditive model—an extension of the geoadditive model proposed by Kammann and Wand [9] and previously used by Diggle et al. [15] and French and Wand [16]. Let the data be denoted by $(x_{i},s_{i},y_{i}),1\leq i\leq n$, where $y_{i}$ is the HIV sero-status of the $i^{th}$ individual, $x_{i}$ and $s_{i}\in R^{2}$ represent the row vector of the covariate values and the geographical location of the $i^{th}$ individual, respectively. Thus, to model the spatial structure for binary data we consider the following model:

(1) $$y_{i}|x_{i},S\left(s_{i}\right)∼\mathrm{Binomial}\left(\pi_{i}\right),\;\mathrm{logit}\left(\pi_{i}\right)=\beta^{T}x_{i}+S\left(s_{i}\right),$$

where β is a vector of parameters related to the different covariates and $S(s_{i})$ represents the geographical component of the model, acting as a surrogate for all unmeasured spatially referenced covariates. $S(s_{i})$, the underlying latent bivariate trend, is modeled using a penalized spline function. One way of specifying the spline function $S(s_{i})$ is to use tensor products of spline bases [17]. Nevertheless, its implementation comes with a price. The number of coefficients increases with the number of knots, and  the tensor products of splines depend on the orientation of the coordinate axes [17]. Alternatively, Powell [18] and Ruppert et al. [17] suggest the use of radial basis functions. Similar to Vandendijck et al. [10], we model the spatial component $S(s_{i})$ through a radial spline basis function of the form:

(2) $$\begin{array}{c} S\left(s\right)=\sum_{k=1}^{K_{s}}u_{k}^{s}\phi_{\tau }\left(\left||s-k_{k}^{s}|\right|\right), \end{array}$$

where $\phi_{\tau }\left(\cdot \right)$ corresponds to a covariance or generalized covariance function similar to what is used in kriging [19,20]. The vector $\left(u_{1}^{s},u_{2}^{s},\cdots ,u_{K}^{s}\right)$ contains the $K_{s}$ unknown coefficients of the radial basis which are penalized for overfitting. The knots $\left\{k_{1}^{s},\cdots ,k_{K_{s}}^{s}\right\}$ are a representative subset of $\left\{s_{1},\cdots ,s_{n}\right\}$ which are used for construction of the basis function. The spatial component in (2) depends on τ, a strictly positive decay parameter which controls how fast the spatial dependence diminishes with increasing distance ||h||. In Equation (2) and throughout, ||·|| denotes the Euclidean distance between two locations.

In model (2) we have to make two choices: the basis function ϕ(·) and the knots $\left\{k_{1}^{s},\cdots ,k_{K_{s}}^{s}\right\}$. In the papers by Kammann and Wand [9] and French and Wand [16], the Matérn covariance function $\phi_{\tau }\left(h\right)=\exp\left(-||h||/\tau \right)\left(1+||h||/\tau \right)$ is used as a radial basis function for the spatial component in (2). In the current paper, we do not restrict ourselves to their choice, but consider a broader range of covariance functions used in kriging [12]. Table 1 summarizes the covariance functions used to model the spatial component (2). A popular way of knot selection in a bivariate dimension is through the efficient space-filling algorithm [21,22]. Regarding the number of knots to be used, Ruppert et al. [17] give a thorough explanation on knot selection. Concurring with Ruppert’s advice, a sensible number of knots is given by K={max20,min(n/4,150)}. Nevertheless, Wand [23] argues that knot specification is of minor relevance when using penalized splines. This is seconded by Ruppert [24], who mention that as the smoothness is controlled by a penalty parameter, the number of knots is of minor importance. Therefore, considering that the number of locations with spatial information is not substantially large in our setting, all different geographical data points are used as knots.

#### 2.2.1. Mixed Model Representation

One appealing feature of the model proposed by Kammann and Wand [9] is the fact that fixing the τ in advance enables one to use a mixed model formulation for fitting, which also holds for the logistic type of model (1). In a mixed model formulation, penalization of the $u_{k}^{s}$ coefficients is analogous to treating them as random effects [17]. Let

$$X={\left[\begin{array}{ccc} 1 & x_{i} & s_{i} \end{array}\right]}_{1\leq i\leq n},\;Z={\left[\begin{array}{c} \phi \left(\left||s_{i}-k_{k}^{s}|\right|\right) \end{array}\right]}_{1\leq i\leq n,1\leq k\leq K},\;\;\;\mathrm{and}\;\;\;\Omega ={\left[\begin{array}{c} \phi \left(\left||k_{k}-k_{k^{{}^{\prime }}}|\right|\right) \end{array}\right]}_{1\leq k\leq K,1\leq k^{\prime }\leq K},$$

then the generalized geoadditive model (1) can be expressed as

(3) logit(π)=Xβ+Zu,

where $u∼N(0,\sigma_{s}^{2}\Omega^{-1})$. The reparametrization $\tilde{Z}=Z\Omega^{-1/2}\;\mathrm{and}\;\tilde{u}=\Omega^{1/2}u$, results in

(4) $$\mathrm{logit}\left(\pi \right)=X\beta +\tilde{Z}\tilde{u},\;\tilde{u}∼N\left(0,\sigma_{u}^{2}I\right).$$

The flexibility to turn a penalized spline smoother into a mixed model enables one to use standard mixed-model software such as R or SAS for fitting the generalized geoadditive model. Furthermore, fitting generalized geoadditive models using mixed model machinery has the advantage that the amount of smoothing is selected automatically. The inclusion of additional linear or non-linear covariates is straightforward. The clustering of observations can be included in the model by adding, for example, a random intercept. In addition, the complex sampling design can be included by using a weighted likelihood, with weights corresponding to the inverse of the sampling probabilities.

#### 2.2.2. Estimation of τ: Two-Stage Iterative Process

Several approaches exist to estimate the range parameter τ. Kammann and Wand [9] proposed to select the maximum distance among the observations, that is, $\hat{\tau }=\max_{1\leq i,j\leq n}\left||s_{i}-s_{j}|\right|$. Kneib [25] uses a similar approach, albeit he re-scales the range parameter such that the correlation among observations at the estimated distance is quite small (e.g., 0.001). More recently, Vandendijck et al. [10] estimated τ via a likelihood-based approach. The choice of τ affects the smoothness of the estimated surface, where large values of τ produce smoother surfaces [16]. Although the smoothness of the estimated surface depends on τ, Zhang and Wang [26] states that the predictions are not substantially affected by the choice of τ. Furthermore, Vandendijck et al. [10] observed that, in fact, the method used by Kammann and Wand [9] does not produce biased prediction, albeit they note that large values of τ inflate the standard errors. The different opinions regarding the estimation and the impact of τ in the predictions highlights the need of a reasonable choice of τ. In this paper, we investigate the estimation of τ in a similar way as done by Vandendijck et al. [10], but in the context of binary data.

Fitting model (1) involves estimating the vector of parameters β and the parameter τ in the covariance function used in (2). Knowing that the likelihood function of (3) is intractable, we use penalized quasi-likelihood (PQL), an approximate inference technique for generalized linear mixed models, to estimate the vector of parameters β. Details of PQL estimation are provided elsewhere [27]. The use of PQL enables us to extend the method proposed by Vandendijck et al. [10] for estimation of τ to the binary context, and here we term it the two-stage iterative PQL-based estimation method. In the first stage the generalized linear mixed model in (3) is estimated fixing τ at its current value, and in the second stage the τ parameter is optimized. This process is iterated until convergence is attained. To be more specific, the estimation method consists of the following steps:

(i) Set initial value ${\hat{\tau }}^{\left(0\right)}$, for the τ parameter.
(ii) Fixing τ at ${\hat{\tau }}^{\left(k\right)}$ in $\phi_{\tau }$ used in (2), fit the generalized linear mixed model in (3) using PQL. This will yield estimates of the variance parameter ${\left({\hat{\sigma }}_{u}^{2}\right)}^{\left(k+1\right)}$, the fixed effects ${\hat{\beta }}^{\left(k+1\right)}$, and random effects ${\hat{u}}^{\left(k+1\right)}$.
(iii) Using ${\left({\hat{\sigma }}_{u}^{2}\right)}^{\left(k+1\right)}$ and ${\hat{\beta }}^{\left(k+1\right)}$, maximize the approximate profile quasi-likelihood function for the variance component considering the pseudo-response $Y_{i}=\eta_{i}^{u}+\left(y_{i}-\mu_{i}^{u}\right)g^{\prime }\left(\mu_{i}^{u}\right)$, where $\eta_{i}^{u}$ is the linear predictor in (4), $\mu_{i}^{u}$ is $E(y_{i}|u)$, and $g(\mu_{i}^{u})$ is the link function. The approximate profile quasi-likelihood function is given by (Breslow and Clayton [27]):
  (5) $$ql\left(\tau \right)\approx -\frac{1}{2}\log\left|V(\tau )\right|-\frac{1}{2}{\left(Y-X\hat{\beta }\right)}^{T}V^{-1}\left(\tau \right)\left(Y-X\hat{\beta }\right)$$
  with respect to τ, $V=ZDZ^{T}+W^{-1}$, and $D=\sigma_{u}^{2}I$, and W is an n×n diagonal matrix with diagonal elements $w_{i}={\left\{var\left(y_{i}|u\right){\left[g^{{}^{\prime }}\left(\mu_{i}^{u}\right)\right]}^{2}\right\}}^{-1}$. The value of τ that maximizes this function is denoted by ${\hat{\tau }}^{\left(k+1\right)}$.
(iv) For k=0,1,…, iterate between steps (ii) and (iii) until the difference between two successive $\hat{\tau }$ values is smaller than a pre-specified tolerance level *c*, so until $|{\hat{\tau }}^{\left(k\right)}-{\hat{\tau }}^{\left(k+1\right)}|<c$.

### 2.3. Model Building

Using model building, we selected the set of covariates and the covariance function. Therefore, model building proceeded in two steps. First, to identify risk factors to be included in the generalized geoadditive model (1), multivariate logistic regression analyses were performed, considering all pairwise interactions of the selected variables presumed to be related to HIV sero-positivity. The selection of candidate variables was done using a backward stepwise selection technique. The search was done using the stepGAIC() function from the gamlss R package (resulting in the set of variables minimizing the Akaike’s information criterion (AIC) value). Second, three model-building scenarios were considered to select the best covariance function: (1) models with only a spatial geographical component, (2) models with both risk factors and spatial geographical component using a two-stage iterative PQL-based estimation method, and (3) models with both risk factor and spatial geographical component but with fixed τ, using the proposal by Kammann and Wand [9]. The choice of the “suitable” covariance function in each of the three scenarios was performed with the aid of AIC, Bayesian information criterion (BIC) and corrected AIC (AICc). Note that in geostatistical models as described in (2–4), AIC fails to properly account for the degrees of freedom in the penalized spline models [10]. Furthermore, Hoeting et al. [28] argue that AIC and BIC ignore the spatial dependence in the data, and advocate the use of AICc [17]. Model performance was assessed by means of confusion matrix, a classification table comparing predicted values against the observed, and the receiver operating characteristic (ROC) curve. The model with the highest balanced accuracy, the average accuracy obtained on either class (positive and negative HIV sero-status), and the highest area under the ROC curve were considered to have good performance. Alternatively, one could use the “regular” accuracy, the number of correctly classified observations overall. Nevertheless, the latter is recommended for balanced data [29]. For this reason, the former was preferred.

All of the analyses in this paper were performed in R software version 3.6.1 [30]. To implement the described estimation method in Section 2.2.2, for estimating the τ parameter, a set of R functions were written. The R codes are provided in the supplementary material.

## 3. Results

### 3.1. Exploratory Analysis

Table 2 gives a summary of participant characteristics by HIV sero-positivity status. HIV prevalence by socio-demographic characteristic varied from 3.73% to 21.15%. The smallest proportion was observed among males and the highest was observed among divorced/widowed persons. Regarding religion, the lowest proportion was observed among those professing other religions (4.07%) and the highest among Protestants (9.93%). HIV prevalence was 10.15% among individuals in urban areas and 6.43% in rural areas. The results also show that the observed HIV prevalence was higher among young individuals living in female-headed households (10.66%). It was also observed that the prevalence was almost two times higher among high-income individuals than among low-income individuals. Prevalence in individuals with more than one sexual partner was almost two times higher when compared to those with only one sexual partner. Additionally, the prevalence among people who reported having ever had any kind of sexually transmitted infection (STI) was considerably larger (12.27%) than among those who reported having never had any kind of STI (7.62%). It can also be noticed that the prevalence of HIV among individuals aged 20–24 years was about two times higher than the prevalence among individuals aged 15–19 years.

#### 3.1.1. Covariance Function

Table 3 shows AICc, AIC, BIC, degrees of freedom (df), and parameter estimates for τ and $\sigma_{u}$ under different covariance functions for the three aforementioned scenarios. In the first scenario, based on AICc and AIC, it can be seen that the models with spherical, exponential, and circular covariance functions appeared to fit the data best. Nevertheless, according to BIC criteria, the model with the Gaussian covariance function outperformed the others. When covariates were included in the model, AICc, AIC, and BIC dropped drastically. Again, it can be observed that AICc and AIC point to models with spherical, exponential, and circular covariance functions as the best ones, while BIC outstandingly points to the model with the Gaussian covariance function as the single best one. Other models that exhibit lower BIC value were the models with the multiquadratic inverse and Matérn covariance function. When τ was fixed, no considerable changes were seen in the AICc and AIC, except for the Gaussian covariance function where it is seen a substantial increase. Therefore, there was no clear winner. Based on BIC, the results point to a model with the Gaussian covariance function as the best fit. Comparing the models where we fixed versus estimated τ, we observe that based on BIC fixing τ to the maximum distance led to a better fit, whereas when we compare using AIC, the two-stage iterative process tended to lead to a better fit. Although AICc and AIC favored spherical, exponential, and circular covariance functions, amongst them no clear winner was observed. For the model with no covariates, AICc, AIC, and BIC were not reported for multiquadratic inverse covariance as convergence was not attained. A covariance function—a member of isotropic covariance functions—that would also be of consideration is the thin plate spline. Nevertheless, all the models failed to converge under this covariance function.

### 3.2. Final Model

#### 3.2.1. Spatial Trend

**Figure 1:**
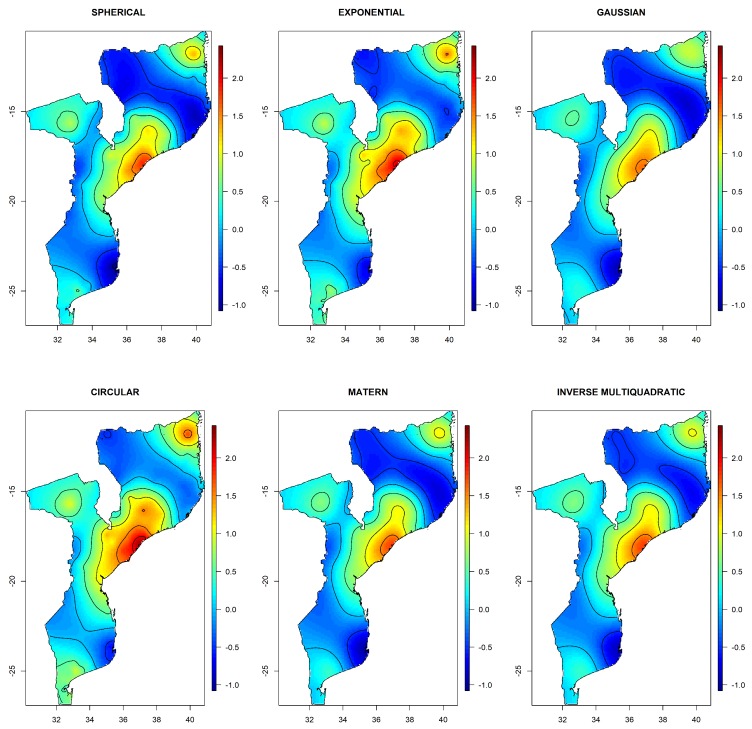
Image plots of the radial spline basis using the two stage-iterative estimation process with different covariance functions. The plotted radial spline basis is on natural log-odds scale. This illustrates the spatial variation of HIV risk in Mozambique.

**Figure A1:**
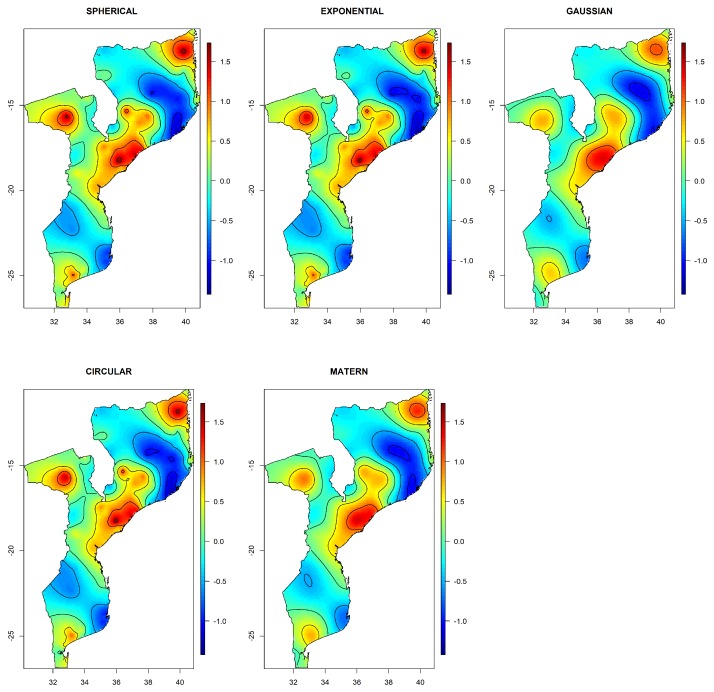
Image plots of the radial spline basis under different covariance functions. The images correspond to the model with only the spatial component. Model is fitted using two-stage iterative estimation process. The radial spline basis is plotted on natural log-odds scale. This illustrates the spatial variation of HIV risk in Mozambique.

Our primary interest was in the spatial distribution of the HIV sero-status. In this subsection we analyze the spatial variation of HIV infection across the country. This was achieved through a visualization of the radial spline basis in a map. We again considered the three scenarios. Figure A1 in the appendix shows the spatial distribution of HIV infection when considering a model with only the spatial component for different covariance functions. Visual inspection suggests that the predictions are insensitive to the choice of the covariance function, indicating robustness to covariance-structure. This was similarly observed for AIC and BIC values, as no clear winner was observed. The maps reveal four different places that exhibit high prevalence, pointing to the effect of a spatial geographical component in the sero-status. Figure 1 shows the spatial variation of HIV after adjustment for the covariates. In this case, the spline component refers to the geographical residual effect, after adjustment for the covariates. As a result, the variation of S(s) under the two-stage iterative adjusted model was less pronounced, illustrating that a substantial part of the variability was explained by the covariates. Again, the spatial variation of HIV under all covariance functions looked very similar, with the Gaussian covariance function showing the smoothest surface. Once more, the similarity of HIV spatial variation across different covariance function indicates that the predictions of HIV prevalence were quite robust to the choice of the covariance function. Further, note that there remains an important geographical trend in HIV prevalence that is not explained by the risk factors in the model.

**Figure A2:**
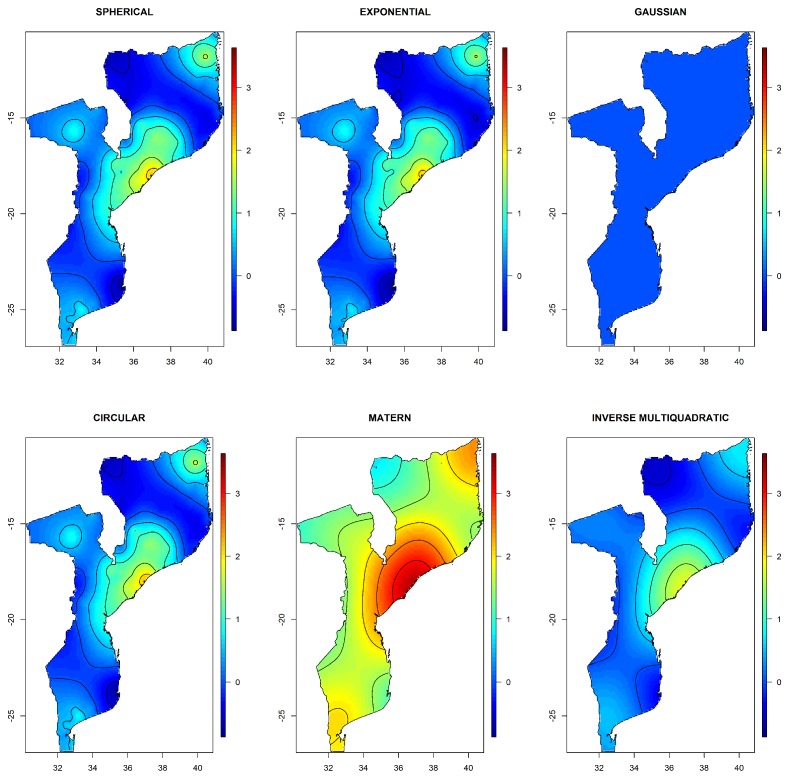
Image plots of the radial spline basis with $\hat{\tau }=\max_{1\leq i,j\leq n}s_{i}-s_{j}$ under different covariance functions. The radial spline basis is plotted on natural log-odds scale. This illustrates the spatial variation of HIV risk in Mozambique.

Results were different when τ was fixed to the maximum distance. Although the model with Gaussian covariance was selected as the best based on BIC (Table 3), the maps in Figure A2 in the Appendix shows that the spatial distribution of HIV reveals an inconsistent behavior, with no variation in S(s). Additionally, the estimated variance of the random effects was almost zero (see Table 3). This points to either estimation instability or a very smooth spatial process. a similar behavior was observed for the Matérn covariance function. This indicates that fixing τ to the maximum distance among the observations is not recommended, as it may lead to parameter instability. For other covariance functions, the spatial distribution of HIV was similar to the one shown by the models fitted using the two-stage iterative process, though the inverse multiquadratic function also showed a much smoother pattern.

The spatial analysis revealed the presence of some trends in the data. After controlling for important covariates, HIV prevalence appeared to be more pronounced in the center of the country, with an odds of becoming HIV infected of about two times higher than in the north-eastern region of the country and three times higher than in the southern region.

#### 3.2.2. Covariate Effects

Table 4 shows the parameter estimates for five different models. The first model is the traditional logistic regression, the second, third, and fourth are generalized geoadditive models fitted using the two-stage iterative estimation process under Gaussian, spherical, and exponential covariance functions, respectively, and the fifth uses the Gaussian covariance function fixing τ to the maximum distance among the observation. From this table it is apparent that parameter estimates from traditional logistic regression and generalized geoadditive model with the Gaussian covariance function when τ was fixed were quite similar. This suggests that fixing τ under the Gaussian covariance function did not lead to any improvement when compared to conventional logistic regression. In fact, this is confirmed by the variance of the random effect, which was almost zero. Additionally, the classification table for this model shows a high imbalance between sensitivity and specificity, also indicating a poor predictive performance (Table A3 and Table A5).

We also observe that the parameter estimates for the models fitted using the two-stage iterative process under the Gaussian, spherical, and exponential covariance function exhibited similar parameter estimates. This again indicates that inference was quite insensitive to the choice of the covariance function.

**Figure A3:**
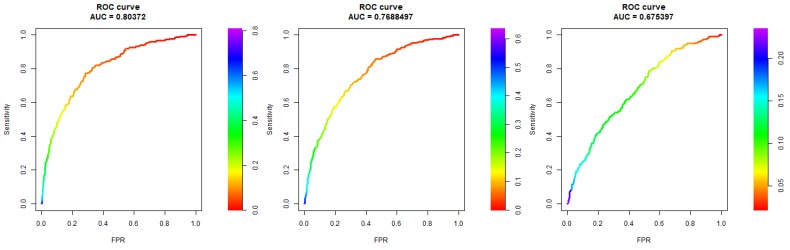
Receiver operating characteristic (ROC) curve for the generalized geoadditive model. The plot on the left corresponds to the ROC curve for the model fitted using the two-stage iterative method. The plot in the middle is for the model fitted with τ fixed to the maximum distance. The plot on the right is for the model fitted with the two-stage iterative method but with no covariates. All three models consider a Gaussian covariance function.

The results from Table 4 reveal no substantial differences between parameter estimates of models fitted using two-stage iterative PQL estimation under the Gaussian, spherical, and exponential covariance functions. Nevertheless, the results in Table 3 point to the model fitted under the Gaussian covariance function as a “suitable” choice. This finding was also supported by the classification tables, where a good balance between sensitivity and specificity was observed by the model fitted under the Gaussian covariance function. Additionally, the accuracy in Table A3 and Table A4, and the ROC curve in Figure A3 also point to the model fitted under two-stage iterative PQL estimation with Gaussian covariance function. For these reasons, the model with the Gaussian covariance function was chosen for inferential purposes.

Results show that HIV sero-positivity was significantly related to sex, where female individuals were about five times more likely to be HIV positive than their male counterparts. Results also show a significant relationship between HIV sero-positivity and religion; individuals with no religion appeared to be almost two times more likely to be HIV positive than those with any religion. We also observed that age was significantly associated with HIV; individuals in the age range of 20–24 were more likely to be HIV positive as compared to adolescent individuals (age range 15–19).

The results further reveal significant interaction effects of place of residence and number of sexual partners, place of residence and sex of household head, wealth index and HIV/AIDS awareness, wealth index and stigma HIV/AIDS, stigma HIV/AIDS and work occupation, and stigma HIV/AIDS and education. We observed that individuals with two sexual partners living in a rural area were about four times more likely to be HIV positive than those with one partner in the same area, and for individuals living in an urban area the chances were almost half when compared to those living in a rural area. It can also be observed that high-income individuals with the highest HIV/AIDS awareness and highest HIV/AIDS stigma beliefs were about 18% less likely to be HIV positive than poor individuals with the highest HIV/AIDS awareness and highest stigma beliefs, whereas the odds of being HIV positive for those with the lowest HIV/AIDS awareness and the lowest HIV/AIDS stigma beliefs was about 13 times higher than for young poor individuals in the same category. Individuals with the highest stigma beliefs who had secondary education were about 67% less likely to be HIV positive as compared to those with no education and with the highest stigma, and those with primary education having highest stigma were about 65% less likely to be HIV positive as compared to those with no education and having highest stigma.

The results also reveal that individuals living in a female-headed household in a rural area are two times more likely to be HIV positive than those living in a male-headed household, whereas individuals living in female-headed households in urban areas were almost 32% less likely to be HIV positive than those in male-headed households. From the results it is also apparent that individuals with three or more partners were three times more likely to be HIV positive than those with only one partner.

## 4. Discussion

Analyses of the spatial distribution of HIV are very crucial, especially in developing countries where resources are scarce, as the results might direct health officials to turn their attention to locations where more resources are needed and build policies to address the burden of HIV/AIDS in specific locations. In this paper, we applied generalized geoadditive models to analyze the spatial distribution of HIV prevalence among young people, by including the geographical location in the estimation process. The inclusion of the geographical location prompted us to use radial spline basis to study the spatial effect of geographical location on the HIV sero-status of the individuals. We extended the method proposed by Vandendijck et al. [10] to the binary setting using PQL estimation. The method allowed us to estimate the effect of both the non-spatial covariates and the decay parameter in the radial basis function describing the spatial component in the generalized geoadditive model.

We observed that the two-stage iterative process PQL was robust to the choice of the covariance function. Further, we noted that the proposal by Kammann and Wand [9] for estimation of the range parameter for some covariance function led to either parameter instability or to a very flat surface (e.g., for Gaussian and Matérn covariance functions no residual variation was observed for the spatial component). Additionally, it is important to note that the Gaussian covariance function resembles a very smooth spatial process. Waller and Gotway [31] argue that such cases rarely occur in practice. Moreover, Davis and Morris [32] state that although it is a valid covariance function, it can often lead to singularities in spatial prediction equations. Thus, results under the Gaussian covariance function should be interpreted with caution. Overall, results based on the two-stage iterative PQL-based estimation method were consistent for the spherical, exponential, and Gaussian covariance functions.

The analyses revealed three main regions with high HIV prevalence, namely southern, central, and the north-eastern regions. This is in agreement with what was observed in the Mozambique AIS report [2]. The high prevalence observed can be explained partly by early marriages and early pregnancies. In the center of Mozambique, the Zambezia and Manica provinces have the highest number of child marriages [33]. The practice of early marriage subjects children to early sexual debut, increasing their lifetime risk for HIV [34]. In addition to early sexual debut, they are also exposed to frequent unprotected sex, generally with men older than them who have multiple sexual partners. In turn, this increases their risk of HIV infection [35,36]. It is also worth emphasizing that in the northern and central parts of the country, before they are forced into early marriage adolescent girls go through initiation rituals, a socio-cultural ceremony where adults pass to young people attitudes and beliefs about sexuality necessary for transitioning to adulthood, which in turn increases their risk of HIV infection [37]. The high prevalence in high-burden regions may partly be explained by the presence of the high-risk population in these areas. A study conducted in Maputo, Beira, and Nampula City, Mozambique found that most of female sex workers were at the ages of 15–24 years [38].

Several socio-demographic, knowledge and attitude-related, behavioral, and biological factors were identified to be associated with HIV infection among individuals aged 15–24 years in Mozambique. In this study, we found that young women were about six times more likely be HIV infected than their male counterparts. Gender inequality has been observed in several studies in sub-Saharan Africa. This can be partly explained by poverty, as poor women are often financially dependent on men, which drives them to early marriage and subsequent low decision-making power. Additionally, oftentimes vulnerable women have little choice but to adopt behaviors that put them at a higher risk of HIV, such as transactional and intergenerational sexual relationships [39]. Furthermore, the large differences of HIV prevalence between young women and men of the same age may also be explained by the fact than women tend to partner with older men, who are much more likely to be HIV positive [40,41]. We found that young individuals aged 20–24 years were more likely to be HIV positive than adolescents aged 15–19 years. This is in line with other studies conducted in Southern Africa [42,43].

The findings indicate that higher education has a protective effect against HIV infection. Generally, it is assumed that educated individuals are more knowledgeable about HIV risk behavior and more likely to be empowered to make decisions about sexual debut, condom negotiation, etc. Therefore, the chances of adopting preventive measures are higher. Additionally, young individuals who know more about HIV/AIDS are more likely to know their HIV status, thus contributing to their health-seeking behaviors and consequently helping to lower the risk of HIV transmission [44]. Our findings are supported by a study done in South Africa showing that adolescent and young women with tertiary education were less likely to be HIV positive [43]. We also found that higher-income people with low HIV/AIDS awareness were more likely to be HIV positive than poor young individuals with the same level of awareness. In fact, there is evidence that wealthier people tend to have more high-risk behaviors, such as multiple sex partners [45,46]. This is an indication of the importance of awareness campaigns on HIV-related matters in the fight against HIV infection.

The analysis further revealed that young individuals in female-headed households in rural areas were more than two times more likely to be HIV positive than young individuals in the male-headed households in rural areas. A different scenario was observed in the urban areas, where the odds of becoming infected for individuals in female-headed households were 32% lower as compared to individuals in male-headed households. In rural areas, many women who become head of household due to the loss of their husband are confronted with many vulnerable experiences, such as loss of property, lack of access to many traditional services, and increased workload to support their basic necessities. Such experiences increase the vulnerability of women, which may result in engaging in practices that increase their risk of HIV. For instance, a study done in Kenya noted that in order to cope with the loss after the death of an adult member, some adolescent daughters engaged in activities that exposed them to higher HIV risk infection, such as early marriage or exchange of sex for money [47]. Although the literature suggests that female-headed households tend to be poor [48], different scenarios may be observed in urban areas as poverty is much more pronounced in rural areas.

Potential limitations of our study include: the geographical locations used were at the level of enumeration areas, and all households in the same enumeration area were assumed to have the same geographical location. When there is a lack of information on the sampled units, Bocci [49] advocates to treat this as a measurement error and assume a distribution on the location inside each enumeration area. The radial basis functions considered in the analysis are only able to describe an isotropic process. In practice, many geostatistical processes are anisotropic. In future, research anisotropy in this type of model should be taken into account. As future research, it would also be interesting to consider the estimation of the decay parameter using a full-likelihood-based estimation approach. Additionally, this was a cross-sectional study, and thus causality of the identified factors cannot be proved. Our approach can also be used for cases where the observed data are counts.

Despite these limitations, the findings are very important and can help health officials and policy makers to design targeted interventions for responding to the HIV epidemic. The results revealed that provinces from the center and the north should be prioritized when allocating resources to fight HIV. Moreover, specific targeted care services and HIV prevention and awareness campaigns are urgently needed among adolescents and youth. To increase awareness, educational campaigns should include messages addressing the specific characteristics of various socio-demographic profiles. Additionally, there is a need to promote education among youth, particularly in rural and low-income areas. Special attention should be given to gender-equity and female empowerment when designing intervention measures.

## Figures and Tables

**Table 1:** Covariance functions $\phi_{\tau }\left(h\right)$ used in the generalized geoadditive model (1).

Exponential	$\exp\left(-\frac{\left||h|\right|}{\tau }\right)$
Gaussian	$\exp\left(-\frac{{\left||h|\right|}^{2}}{\tau^{2}}\right)$
Spherical	$\left(1-\frac{3}{2}\frac{\left||h|\right|}{\tau }+\frac{1}{2}\frac{{\left||h|\right|}^{3}}{\tau^{3}}\right)I_{\left||h|\right|<\tau }$
Matérn	$\exp\left(-\frac{\left||h|\right|}{\tau }\right)\left(1+\frac{\left||h|\right|}{\tau }\right)$
Circular	$1-\frac{2}{\pi }\left(\vartheta \sqrt{1-\vartheta^{2}}+\arcsin\vartheta \right)$, with $\vartheta =\min\left(\frac{\left||h|\right|}{\tau },1\right)$
Inverse Multiquadratic	$\frac{1}{\sqrt{1+\frac{{\left||h|\right|}^{2}}{\tau }}}$

**Table 2:** Distribution of HIV prevalence by demographic factors. PCA: principal component analysis; STI: sexually transmitted infection.

	HIV Positive, n (%)	HIV Negative, n (%)	Study Population, n (%)
**Demographic factors**			
Sex			
*Male*	55 (3.73)	1420 (96.27)	1475 (43.31)
*Female*	215 (11.13)	1716 (88.87)	1931 (56.69)
Age			
*15–19*	86 (4.99)	1636 (95.01)	1722 (50.56)
*20–24*	184 (10.93)	1500 (89.07)	1684 (49.44)
Religion			
*Catholic*	75 (6.53)	1074 (93.47)	1149 (33.77)
*Muslim*	41 (7.28)	522 (92.72)	563 (16.55)
*Protestant*	108 (9.93)	980 (90.07)	1088 (31.98)
*No religion*	39 (9.05)	392 (90.95)	431 (12.67)
*Other*	7 (4.09)	164 (95.91)	171 (5.03)
Education			
*None*	42 (9.46)	402 (90.54)	444 (13.04)
*Primary*	166 (8.34)	1825 (91.66)	1991 (58.47)
*Secondary/Higher*	61 (6.29)	909 (93.71)	970 (28.49)
Sex of household head			
*Male*	162 (6.77)	2230 (93.23)	2392 (70.25)
*Female*	108 (10.66)	905 (89.34)	1013 (29.75)
Marital Status			
*Divorced/Widowed*	11 (21.15)	41 (78.85)	52 (1.53)
*Married*	148 (9.36)	1434 (90.64)	1582 (46.45)
*Never married*	111 (6.26)	1661 (93.74)	1772 (52.03)
Wealth index			
Poor	50 (4.61)	1034 (95.39)	1084 (31.83)
Rich	220 (9.47)	2102 (90.53)	2322 (68.17)
Media exposure			
*Lowest*	28 (7.76)	333 (92.24)	361 (10.63)
*Second quarter*	88 (7.12)	1148 (92.88)	1236 (36.41)
*Third quarter*	70 (8.95)	712 (91.05)	782 (23.03)
*Highest*	84 (8.27)	932 (91.73)	1016 (29.93)
Occupation			
*Not working*	120 (7.49)	1482 (92.51)	1602 (47.21)
*Working*	148 (8.26)	1643 (91.74)	1791 (52.79)
Place of residence			
*Urban*	139 (10.15)	1231 (89.85)	1370 (40.22)
*Rural*	131 (6.43)	1905 (93.57)	2036 (59.78)
**Proximate factors**			
HIV/AIDS Stigma			
*Lower than the PCA average*	112 (8.26)	1244 (91.74)	1356 (39.81)
*Higher than the PCA average*	158 (7.71)	1892 (92.29)	2050 (60.19)
Know someone with AIDS			
*No*	170 (7.46)	2109 (92.54)	2279 (66.97)
*Yes*	100 (8.90)	1024 (91.10)	1124 (33.03)
Previously tested for HIV/AIDS			
*No*	152 (6.37)	2235 (93.63)	2387 (70.23)
*Yes*	116 (11.46)	896 (88.54)	1012 (29.77)
**Sexual behavioral factors**			
STI, genital sore/ulcer or discharge			
*No*	235 (7.62)	2850 (92.38)	3085 (93.34)
*Yes*	27 (12.27)	193 (87.73)	220 (6.66)
Multiple Partners			
1	63 (6.34)	931 (93.66)	994 (36.22)
2	79 (11.40)	614 (88.60)	693 (25.26)
3+	102 (9.65)	955 (90.35)	1057 (38.52)

**Table 3:** Akaike information criterion (AIC), corrected AIC (AICc), and Bayesian information criterion (BIC) values for the three model-building scenarios and for different covariance functions, together with the parameter estimates for τ and $\sigma_{u}$.

	AICc	AIC	BIC	df	$\hat{\tau }$	${\hat{\sigma }}_{u}$
	No covariates, two-stage iterative					
Spherical	1368.11	1466.92	1756.50	49.42	16.99	2.62
Exponential	1367.49	1466.81	1757.91	49.68	17.00	3.23
Gaussian	1429.82	1480.52	1629.09	25.36	1.45	0.73
Circular	1368.11	1466.94	1756.58	49.43	17.00	2.85
Matérn	1415.19	1476.69	1656.92	30.76	0.80	0.89
Multiquadratic Inverse						
	With covariates, two-stage iterative					
Spherical	1147.22	1264.79	1609.361	58.81	4.12	0.925
Exponential	1145.37	1266.03	1619.66	60.36	17.00	2.427
Gaussian	1179.78	1272.23	1543.16	46.24	1.83	0.72
Circular	1145.97	1265.76	1616.81	59.92	13.91	1.92
Matérn	1168.34	1269.51	1566.01	50.61	0.94	0.83
Multiquadratic Inverse	1173.65	1271.19	1557.03	48.79	2.07	0.98
	With covariates, τ fixed to max distance					
Spherical	1146.25	1266.20	1617.74	59.99	17.20	1.97
Exponential	1145.38	1266.03	1619.64	60.35	17.20	2.44
Gaussian	1265.74	1323.71	1493.63	29.00	17.20	0.00
Circular	1146.18	1266.24	1618.07	60.05	17.20	2.14
Matérn	1197.84	1280.46	1522.60	41.33	17.20	13.77
Multiquadratic Inverse	1196.91	1278.38	1517.13	40.75	17.20	1.65

**Table 4:** Parameter estimates for multivariate logistic regression and generalized geoadditive model (logistic regression adjusted for spatial effect). The parameter estimates $\hat{\beta }$ are on natural log-odds scale, and between brackets standard errors (s.e.) are reported. Statistically significant coefficients are emphasized.

Covariates	Logistic Regression	Gaussian	Spherical	Exponential	Gaussian-Tau Fixed
	$\hat{\beta }$ (s.e.)	$\hat{\beta }$ (s.e.)	$\hat{\beta }$ (s.e.)	$\hat{\beta }$ (s.e.)	$\hat{\beta }$ (s.e.)
Intercept	**−6.176 (0.627)**	**−9.439 (2.594)**	**−9.492 (2.629)**	**−8.349 (2.633)**	**−6.743 (2.282)**
Sex (ref = Male)					
Female	**1.615 (0.212)**	**1.704 (0.216)**	**1.725 (0.217)**	**1.725 (0.217)**	**1.617 (0.213)**
Age(ref = 15–19)					
20–24	**0.506 (0.168)**	0.545 (0.172)	**0.548 (0.172**)	**0.549 (0.172)**	**0.508 (0.168)**
Education (ref = No education)					
Primary	0.228 (0.331)	0.177 (0.342)	0.159 (0.343)	0.156 (0.342)	0.241 (0.330)
Secondary/higher	−0.774 (0.471)	−0.642 (0.484)	−0.663 (0.486)	−0.668 (0.485)	−0.758 (0.471)
Religion (ref = Catholic)					
Muslim	−0.277 (0.243)	−0.142 (0.264)	−0.074 (0.265)	−0.068 (0.265)	−0.36 (0.257)
No religion	**0.641 (0.249)**	**0.569 (0.269)**	**0.576 (0.270)**	**0.561 (0.270)**	**0.714 (0.265)**
Protestant	**0.495 (0.194)**	**0.595 (0.214)**	**0.619 (0.215)**	**0.614 (0.215)**	**0.584 (0.214)**
Other	−0.566 (0.430)	−0.266 (0.446)	−0.253 (0.448)	−0.26 (0.448)	−0.469 (0.442)
Wealth index (ref = Poor)					
Rich	**2.555 (0.476)**	**2.623 (0.492)**	**2.515 (0.488)**	**2.508 (0.487)**	**2.628 (0.481)**
Residence (ref = Urban)					
Rural	−1.099 (0.327)	**−0.827 (0.336)**	**−0.85 (0.337)**	**−0.843 (0.337)**	**−1.105 (0.327)**
Occupation (ref = Not working)					
Working	−0.152 (0.240)	−0.223 (0.246)	**−0.204 (0.247)**	−0.198 (0.247)	−0.143 (0.240)
Sex of the household head (ref = Male)					
Female	−0.537 (0.398)	−0.399 (0.404)	−0.395 (0.404)	−0.397 (0.404)	−0.528 (0.400)
HIV/AIDS awareness (ref = Lowest)					
Highest	1.609 (0.398)	**1.801 (0.408)**	1.769 (0.407)	**1.764 (0.406)**	1.629 ( 0.399 )
HIV/AIDS stigma (ref = Lowest)					
Highest	1.403 (0.523)	**1.106 (0.537)**	0.979 (0.537)	0.976 (0.537)	1.397 (0.524)
Sexual partners (ref = 1)					
2	0.468 (0.330)	0.621 (0.344)	0.621 (0.345)	0.629 (0.345)	0.464 (0.333)
3+	**0.955 (0.308)**	**1.175 (0.326)**	**1.18 (0.327)**	**1.192 (0.327)**	**0.970 (0.316)**
Wealth index × HIV/AIDS stigma					
Rich × Highest	**−1.442 (0.440)**	**−1.102 (0.449)**	**−0.983 (0.449)**	**−0.985 (0.449**)	**−1.445 (0.440)**
Wealth index × HIV/AIDS awareness					
Rich × Highest	**−1.697 (0.439)**	**−1.728 (0.450)**	−1.673 (0.449 )	**−1.666 (0.448)**	**−1.715 (0.440)**
Residence ×Sexual partners					
Rural × 2	**0.935 (0.411)**	**0.829 (0.421)**	**0.813 (0.422)**	**0.802 (0.422)**	**0.935 (0.412)**
Rural × 3+	−0.569 (0.407 )	**−0.757 (0.420)**	**−0.729 (0.422)**	**−0.735 (0.422)**	**−0.627 (0.412)**
Residence × Household head					
Rural × Female	**1.361 (0.360)**	**1.326 (0.366)**	**1.332 (0.367)**	**1.335 (0.366)**	**1.385 (0.361)**
Household head × Sexual partners					
Female × 2	−0.731 (0.437)	−0.756 (0.443)	−0.723 (0.444)	−0.713 (0.444)	−0.717 (0.438)
Female × 3+	**0.845 (0.420)**	0.807 (0.427)	0.795 (0.429)	0.794 (0.429)	**0.852 (0.422)**
HIV/AIDS stigma × Occupation					
Highest × Working	**0.692 (0.317)**	**0.724 (0.326)**	**0.719 (0.328)**	**0.707 (0.327)**	**0.697 (0.318)**
HIV/AIDS stigma× Education					
Highest × Primary	**−1.037 (0.430)**	**−1.125 (0.441)**	**−1.117 (0.444)**	**−1.106 (0.443)**	**−1.028 (0.430)**
Highest × Secondary/Higher	−0.243 (0.559 )	−0.483 (0.571)	−0.458 (0.574)	−0.445 (0.574)	−0.206 (0.560)
Longitude		0.069 (0.058)	0.066 (0.058)	0.038 (0.058)	0.020 (0.051)
Latitude		−0.019 (0.031)	−0.029 (0.032)	−0.021 (0.032)	0.013 (0.028)

## Appendix A

**Table A1:** Description of the variables used in the study.

Variable Name	Discription
**Outcome variable**	
HIV sero-status	Coded as 1 if the respondent is HIV positive and 0 otherwise.
**Demographic factors**	
Age group (ref = 15–19)	respondent age group: 15–19: 0; 20–24: 1
Gender (ref = Male)	Male: 0; Female: 1
Residence (ref = Urban)	Respondent living in Rural area: 1; Urban area: 0.
Education level(ref = None)	Highest educational attainment None: 0; Primary: 1; Secondary/higher education: 3.
Sex of the household head (ref = Female)	Respondent living at female-headed household at the time of the survey: 0; Not living at a female-headed household: 1.
Religion (ref = No religion)	Religious affiliation: Catholic: 4; Protestant: 3; Muslim: 2; Other religion: 1; and No religion: 0.
Marital Status (ref = Never married)	Marital status classified into three categories: Never married: 0; Married/living together: 1; Divorced/widowed: 3.
Occupation	Respondent is currently working: 1; Not working: 0.
Wealth index (ref = Richer and richest)	Composite measure of household’s cumulative living standard derived from information on household ownership using PCA, where the PCA scores are classified into wealth quintiles.
Media exposure (ref = Lowest)	Average media exposure index. The PCA scores are classified into quartiles, the lowest being equivalent to lowest media exposure.
**Proximate HIV/AIDS factors**	
Awareness HIV/AIDS (ref = Lowest)	Average HIV/AIDS awareness index for the respondents. The PCA scores are classified into two groups: lower than the PCA score average and higher than the PCA score average.
Stigma HIV/AIDS (ref = Lowest)	Average HIV/AIDS stigma score. The PCA scores were classified into two groups: lower than the PCA score average and higher than the PCA score average.
Knows someone with AIDS (ref = No)	Respondent knows anyone with AIDS: 1; Does not know: 0.
Previously tested for HIV (ref = No)	Respondent previously tested: 1; Not previously tested: 0.
**Sexual behavior factors**	
Sexual partners (ref = One partner)	Number of sexual partners in the last 12 months. The variable is categorized into three categories: One partner: 0; Two partners: 1; More than three partners: 2.
Alcohol consumption (ref = Did not drink)	How often the respondent had alcohol in the last 12 months. Did not drink: 0; Once a month: 1; Twice or more: 2.
**Biological factors**	
STI, genital sore/ulcer, or discharge (ref = No)	Respondent had any STI, genital sore/ulcer, or discharge in the last 12 months

**Table A2:** Questionnaire items used to derive media exposure index, HIV/AIDS awareness index, and HIV/AIDS stigma index.

Media Exposure	HIV/AIDS Stigma
Frequency of reading newspaper or magazine	Willing to care for relative with AIDS
Frequency of listening to radio	Person with AIDS allowed to continue teaching
Frequency of watching television	Would buy vegetables from vendor with AIDS
Did you use the internet last week?	
**HIV/AIDS awareness**	
Reduce chance of HIV/AIDS by always using condoms during sex	
Reduce chance of HIV/AIDS by having only one sexual partner	
HIV/AIDS can be transmitted through mosquito bites	
HIV/AIDS can be transmitted by sharing food with someone that has AIDS	
HIV/AIDS can be transmitted through pregnancy	
HIV/AIDS can be transmitted through breastfeeding	
HIV/AIDS can be transmitted through delivery	

**Table A3:** Confusion matrix. Cut-off point = 0.09.

	Predicted					
	Two-Stage Iterative		Fixed		Two-Stage Iterative (No Covariates)	
Observed	Negative	Positive	Negative	Positive	Negative	Positive
Negative	1680 (0.713)	675 (0.287)	1570 (0.667)	785 (0.333)	1172 (0.498)	1183 (0.502)
Positive	53 (0.226)	181 (0.774)	64 (0.274)	170 (0.726)	63 (0.269)	171 (0.731)
Balanced Accuracy	0.7434		0.6966		0.6142	

**Table A4:** Confusion matrix. Cut-off point = 0.079.

Predicted						
	Two-Stage Iterative		Fixed		Two-Stage Iterative (No Covariates)	
Observed	Negative	Positive	Negative	Positive	Negative	Positive
Negative	1574 (0.668)	781 (0.332)	1462 (0.621)	893 (0.379)	1013 (0.43)	1342 (0.57)
Positive	45 (0.192)	189 (0.808)	55 (0.235)	179 (0.765)	46 (0.197)	188 (0.803)
Balanced Accuracy	0.7380		0.6929		0.6168	

**Table A5:** Confusion matrix. Cut-off point = 0.5.

Predicted				
	Two-Stage Iterative		Fixed	
Observed	Negative	Positive	Negative	Positive
Negative	2342 (0.994)	13 (0.006)	2348 (0.997)	7 (0.003)
Positive	220 (0.94)	14 (0.06)	229 (0.979)	5(0.021)
Balanced Accuracy	0.52715		0.50920	
